# Impact of COVID-19 Pandemic on Life-Space Mobility of Older Adults Living in Brazil: REMOBILIZE Study

**DOI:** 10.3389/fpubh.2021.643640

**Published:** 2021-04-09

**Authors:** Monica R. Perracini, Juleimar Soares Coelho de Amorim, Camila Astolphi Lima, Alexandre da Silva, Francis Trombini-Souza, Daniele Sirineu Pereira, Paulo Henrique Silva Pelicioni, Etiene Duim, Patricia Parreira Batista, Renato Barbosa dos Santos, Maria do Carmo Correia de Lima, The REMOBILIZE Research Network (CANSORT-SCI)

**Affiliations:** ^1^Master's and Doctoral Program in Physical Therapy, Universidade Cidade de São Paulo, São Paulo, Brazil; ^2^Master's and Doctoral Programs in Gerontology, Faculty of Medical Sciences, Universidade Estadual de Campinas, Campinas, Brazil; ^3^Physiotherapy Undergraduate Program, Instituto Federal do Rio de Janeiro, Rio de Janeiro, Brazil; ^4^Department of Collective Health, Faculdade de Medicina de Jundiaí, Jundiaí, Brazil; ^5^Master's and Doctoral Program in Rehabilitation and Functional Performance, Universidade de Pernambuco, Petrolina, Brazil; ^6^Department of Physical Therapy, Universidade Federal de Minas Gerais, Belo Horizonte, Brazil; ^7^Division of Health Sciences, School of Physiotherapy, University of Otago, Dunedin, New Zealand; ^8^Department of Diagnostic and Ambulatory Medicine, Hospital Israelita Albert Einstein, São Paulo, Brazil

**Keywords:** participation, COVID-19, social distancing, health status disparities, well-being

## Abstract

**Background:** The COVID-19 pandemic hit Brazil in a scenario of substantial socioeconomic and health inequalities. It is unknown the immediate impact of social restriction recommendations (i.e., lockdown, stay-at-home) on the life-space mobility of older people.

**Objective:** To investigate the immediate impact of COVID-19 pandemic on life-space mobility of community-dwelling Brazilian older adults and examine the social determinants of health associated with change in life-space mobility.

**Design:** Baseline data from a prospective cohort study (REMOBILIZE Study).

**Setting:** Community.

**Subject:** A convenience snowball sample of participants aged 60 and older (*n* = 1,482) living in 22 states in Brazil.

**Methods:** We conducted an online and phone survey using an adapted version of the Life-Space Assessment (LSA). Linear regression models were used to investigate social determinants of health on the change in LSA score.

**Results:** Regardless of their gender and social determinants of health, participants showed a significant reduction in life-space mobility since COVID-19 pandemic outbreak. Life-space mobility reduction was higher among black individuals, those living alone and aged between 70 and 79. Other variables associated with change in life-space mobility, to a lesser extent, were sex, education and income.

**Conclusion:** Social restriction measures due to pandemic caused substantial reduction in older adults' life-space mobility in Brazil. Social inequalities strongly affected vulnerable groups. Concerted actions should be put in place to overcome the deterioration in life-pace mobility amongst these groups. Failure in minimizing health inequalities amplified by the pandemic may jeopardize the desired achievements of the Decade of Healthy Aging.

## Introduction

Experts agree that older people are the group most affected by the COVID-19 pandemic (1, 2). Worldwide, more than 66% of adults aged 70 years and over have underlying conditions and are at higher risk for severe disease, which may result in hospitalization and death (3). Social restriction recommendations (i.e., lockdowns, social distancing, stay-at-home orders) have been set up as population-level measures to suppress community transmission of COVID-19 (4). Although these measures were adopted worldwide, how different groups of older people adapted their life-space mobility to these new circumstances (5) is still uncertain. Varying housing conditions, social inequalities, and governments' response policies may have affected how older people moved around since the COVID-19 pandemic (6–8).

Life-space mobility is not a new concept (9); it corresponds to how people engage in, maintain social relationships and roles, and participate in meaningful activities within their communities (10). It is recognized as a practical measure to capture older people's functional ability for moving around in their environments in a specific period of time (11). Restriction of life-space mobility occurs due to a combination of losses in individuals' intrinsic capacity, limited personal resources, and difficulty dealing with environmental challenges, resulting in potentially health adverse outcomes (9).

Restrictions in life-space mobility (12, 13) and in active aging scores (12) were observed in community-dwelling older people during the COVID-19 pandemic. Active aging was evaluated using a novel scale that encompasses older people's striving for well-being through activities pertaining to their goals, abilities, and opportunities (14). Declines in life-space mobility and active aging unsurprisingly coincided, since social restriction policies may have reduced opportunities for several out-of-home activities (12).

Foreseen consequences of constriction in life-space mobility observed in previous studies are decreased levels of physical activity (15, 16), higher prevalence of depressive symptoms (17), cognitive decline (18, 19), poor physical capacity (11), obesity (6), and increased risk for developing frailty (9). Particularly, inactivity related to deconditioning (20, 21) increases the risk of health deterioration associated with chronic non-communicable diseases (21, 22) and may accelerate the loss of muscle mass and muscle strength, along with the accumulation of body fat. Ultimately, inactivity results in poorer overall health (23).

Social inequalities may contribute to the negative impact of social restriction recommendations on life-space mobility since COVID-19 pandemic, particularly for older people living in low-resource settings (24). Previous studies have shown that lower life-space mobility scores were associated with female sex, low educational level, insufficient income (6, 7, 11), and poor physical and social environments (7). Underlying inequalities of gender, race/ethnicity, income, and residential segregation may expose vulnerable groups of older people to negative consequences of the COVID-19 pandemic (25).

Our hypothesis is that levels of life-space mobility throughout the pandemic will exhibit different trajectories according to social determinants. Investigating how social factors influence life-space mobility in this unique period can help to develop interventions needed to deal with the deleterious effects of the COVID-19 pandemic on health systems, individuals, and their families (20, 26, 27). Therefore, this study (i) investigated the immediate impacts of COVID-19 pandemic on the life-space mobility of community-dwelling Brazilian older adults; and (ii) examined the social determinants of health associated with change in life-space mobility.

## Methods

### Study Design, Setting, and Participants

We used baseline data from the REMOBILIZE study, which involved a cohort survey to investigate life-space mobility throughout the course of the COVID-19 pandemic and used a task-force research network for a 12-month follow-up period. We surveyed a convenience snowball sample of older adults aged 60 and older (*n* = 1,482) living in 22 (82%) states in Brazil, using the online platform SurveyMonkey^®^. We used social media (Facebook^®^ and Instagram^®^) and WhatsApp^®^ to recruit participants. A website was set up to reinforce the legitimacy of the study and to provide a central address for respondents to contact the research team. We contacted community leaders and allied health professionals working in vulnerable regions to include participants with different educational and income levels, ethnicities, and genders. We excluded bedridden participants and older adults living in long-term care facilities. Older adults with cognitive decline or who were unable to answer interview questions due to visual or other difficulties, such as digital illiteracy, were helped by a proxy—either a family, friend, or formal caregiver. We conducted data collection between May 18th, 2020 and July 4th, 2020, and participants took approximately 30 min to complete the survey.

The Ethical Research Committee of Universidade Cidade de São Paulo approved all research procedures (protocol number 4.032.523). A consent form was included in the online survey questionnaire as well as given in interviews conducted by telephone. Participants consented or declined to participate in the study by selecting an on-screen button.

### Measures

#### Life-Space Mobility

Life-space mobility was assessed using a Brazilian Portuguese version of the Life-Space Assessment (LSA; (28). The LSA comprises five life-space levels: (1) rooms other than the bedroom, (2) areas outside the house (i.e., porch, deck, yard, hallway of an apartment building or garage), (3) neighborhood other than own yard or apartment building, (4) outside the neighborhood, but within town; and (5) places outside one's own town.

At the baseline, participants were asked about the places they reached both before the COVID-19 pandemic and a week before evaluation (since the pandemic period). For each level, participants were asked how often within the week they attained that level (less than once a week, one to three times a week, four to six times a week, or daily) and whether they needed any help to move to that level (without assistive device or assistance, with an assistive device, or with personal assistance). In the original instrument, displacement is evaluated in the previous 4 weeks, and the respondent is asked to appraise how many times a week he/she attained that place. As most participants in our study answered the questionnaire online without the assistance of an interviewer, we chose to ask about the last week to avoid misinterpretation. Life-space mobility questionnaires have been applied in different timeframes according to specific populations and circumstances (9, 29).

A composite score is calculated by multiplying each life-space level reached by the degree of independence and frequency (30). Score range from 0 to 120 points; higher scores represent greater mobility in space (11, 28). The original instrument demonstrated a reproducibility of 0.97 (95% CI 0.95–0.98). A moderate negative correlation between LSA scores and accelerometry was observed (−0.63, 95% CI −0.74–−0.40) (28).

#### Social Factors and Comorbidities

Independent variables were gender, age group (60–69, 70–79, and ≥80 years), self-report of skin color/race/ethnicity categorized according to official Brazilian classification (white, black, *pardo, amarelo*, or indigenous), marital status (single, married, divorced, widowed), and education level (illiterate, 1–4 years, 5–8 years, and ≥9 years of schooling), living alone (yes/no), income level presented as the minimum wage per month guaranteed by law in Brazil (<1, 2–3, 4–7, 8–10, and >10 minimum wage salaries), employment (active, inactive, or unemployed), receiving pension (yes/no), and reported comorbidities using the Functional Comorbidity Index (FCI) questionnaire (31). The FCI is composed of 18 comorbidities related mainly to physical function. Comorbidities were summed up, and older adults with two or more diseases were considered to have multimorbidity (32).

#### Reported Social Restriction

Adherence to social restriction measures was captured using a five-point Likert scale question: “Do you think you are following the recommendations for social restriction measures?” Possible responses were strongly agree, partially agree, indifferent, partially disagree, and totally disagree. We also asked participants, “What best describes you at this moment?” Possible responses were “living a normal life, nothing has changed in my daily routine;” “being careful, but going out for work, visiting family members or other activities;” “going out only when it is inevitable, such as for food supplies, health-related appointments, or to the drugstore;” “I have been receiving family members, friends, and delivery services;” “completely isolated, not going out at all;” “going out just for walking/jogging;” and “other.”

### Data Storage and Availability

The raw data from the baseline survey were exported from the SurveyMonkey^®^ platform. During this stage, incomplete questionnaires were identified and excluded. Two independent researchers checked the complete submissions to search for possible duplicates or inconsistent data, such as missing consent or date of birth, bedridden status, and residents of long-term care facilities. Searches for zip codes were also conducted. A final anonymized data set was created with all eligible participants. We used only cases for which the full information for all variables of interest for the present study was available. The variables in this dataset have not been recoded or imputed. The data and codebook are available at: https://datadryad.org/stash/share/Rj8_jEF6Tg40YBJolay_Hymqn_Azh3QedL1mPQX9kyg.

### Statistical Analyses

Descriptive analyses were performed, both for the total sample and based on the investigated outcomes, using proportions and means (and standard deviation). LSA scores before and since the COVID-19 pandemic were computed for each level (home, outside home, neighborhood, town, and beyond town), and for the composite score. The difference in total scores before and since pandemic was presented as a delta (Δ LSA). We verified whether the data set (LSA score and Δ LSA) in each group analyzed had a normal distribution using the Shapiro-Wilk test. We used the non-parametric Wilcoxon test for paired data to compare the composite score, the score for each level, and the delta score. Univariate analysis of the associations between independent variables and changes in LSA scores was evaluated by Wilcoxon signed-rank (dichotomous variables) and Kruskal-Wallis tests (categorical variables).

To examine whether social determinants were associated with the Δ LSA, we used crude and adjusted multiple linear regression analyses. Social factors, comorbidities, and adherence to social restriction were selected as multivariate adjusted model variables. A backward stepwise method was used to obtain the final model. The results of multiple linear regression are reported as regression coefficients (β) and 95% confidence intervals (95% CI). We evaluated the adequacy of the model by a set of statistics. The statistics' adjusted *R*^2^ scores were used to verify the percentage of variance related to the decrease in Δ LSA explained by the model. The Durbin-Watson statistic was used to verify the assumption that the residuals were not correlated. We also tested for multicollinearity in the final model, according to variance inflation factors (VIF > 1.10). To evaluate whether the residuals had a normal distribution, the following graphs were performed: standardized regression residuals by standardized regression-predicted values, histogram of frequencies of standardized regression residuals, and a quantiles-quantile graph (QQ plot).

Stata 14.0 (Stata Corporation LLC, College Station, TX) was used for statistical analyses, and the level of statistical significance was set at *p* < 0.05.

## Results

**Figure 1:**
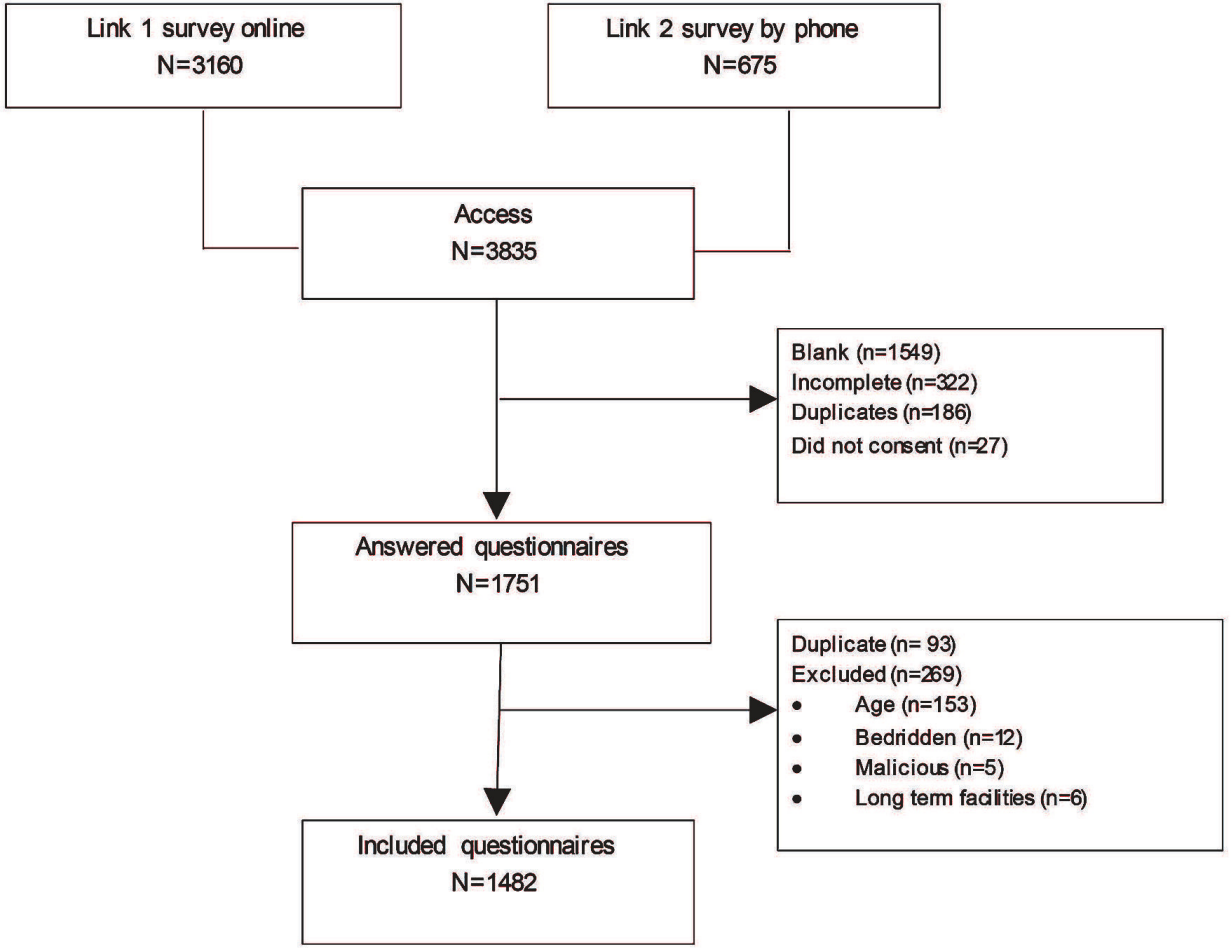
Study flowchart.

After removing incomplete and duplicate questionnaires, 1,482 participants were included who provided all information requested for the study (Figure 1). Seven hundred and ninety nine respondents (53.9%) declared that they had answered the questionnaire by themselves; 534 (36.0%) respondents had the support of a family member, friend, or others to answer the survey; and 149 (10.1%) respondents were proxies.

Mean age was 70.0 (SD 8.14) years old. Seventy three point nine percentage were women, 53.7% were married, 61.7% reported white ethnicity, and 60.9% had 9 or more years of schooling. Approximately half of the participants reported two or more diseases, and more than 80% totally agreed that they were following social restriction measures. Participants' sociodemographic characteristics, comorbidities, and reported adherence to social restriction measures are described in Table 1.

**Table 1:** Social determinants, multimorbidity and responses to social restriction measures among community-dwelling older people between May and July 2020 (33).

**Characteristic**	***N* = 1,482 (%)**
Female Gender	1,096 (73.9)
Age groups (years)	
60–69	831 (56.1)
70–79	420 (28.4)
80 and over	229 (15.5)
Ethnicity	
White	915 (61.7)
Black	100 (6.8)
“*Pardo*”	439 (29.6)
“Amarelo”	23 (1.6)
Indigineous	5 (0.3)
Marital status	
Single	152 (10.3)
Married	796 (53.7)
Divorced	184 (12.4)
Widowed	350 (23.6)
Living alone	256 (17.3)
Educational level (years of schooling)	
Illiterate	117 (7.9)
1–4	282 (19.0)
5–8	181 (12.2)
9 or more	902 (60.9)
Income (minimum wage salary)^a^	
<1	512 (34.5)
2–3	413 (27.9)
4–7	267 (18.1)
8–10	114 (7.7)
10 or more	176 (11.9)
Employment	
Active	545 (36.8)
Inactive	836 (56.4)
Unemployed	101 (6.8)
Pension (yes)	1,215 (82.0)
Multimorbidity (two or more)^b^	841 (56.8)
Following social restriction measures	
Strongly and partially disagree	47 (3.2)
Partially agree	201 (13.6)
Totally agree	1,234 (83.3)
Social restriction behavior since pandemic	
Living without any routine change	42 (2.8)
Being careful, but leaving home to work and visit relatives	169 (11.4)
Leaving home for unavoidable matters (e.g., groceries, pharmacy)	693 (46.8)
Restricted at home, but receiving visits (relatives, friends, deliveries)	132 (8.9)
Restricted at home and not receiving visits	432 (29.1)
Going out for a walk, as exercise	8 (0.5)
missing data	6 (0.4)

a Brazilian minimum wage salary 1,045.00 BRL (corresponding to 189.3 USD; 1st May 2020)

b *Multimorbidity included stroke, Parkinson's disease, arthritis, osteoporosis, urinary and fecal incontinence, acute myocardial infarction, intestinal and depressive disease, anxiety, visual and hearing impairment, spine, overweight, hypertension and dizziness*.

**Figure 2:**
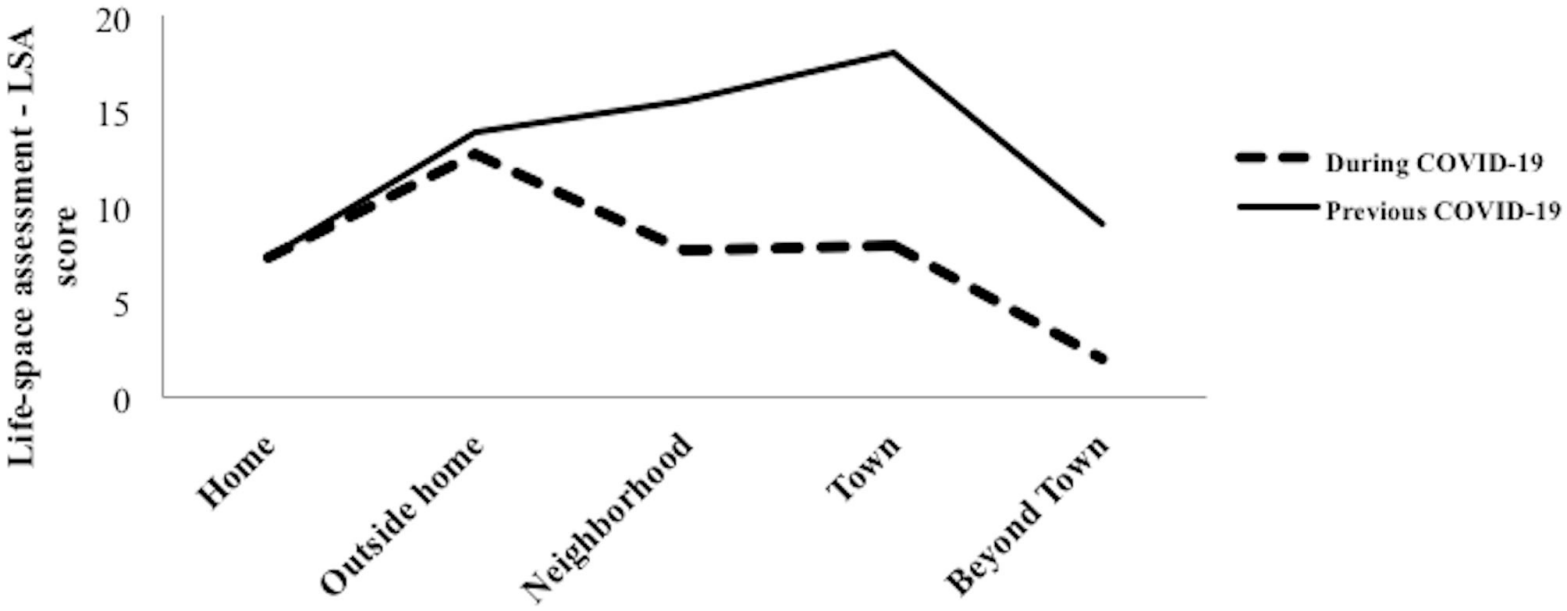
Life-space mobility scores in each level before and since COVID-19 pandemic.

The mean LSA score before the COVID-19 pandemic was 64.0 (SD 26.0) and mean LSA score since the pandemic was 37.8 (SD 22.1), and the Δ was −26.2 (SD 25.0). A significant reduction was observed in LSA scores from Level 2 up to Level 5 (*p* < 0.001; see Figure 2 and Table 2).

**Table 2:** Life-space assessment (LSA) according to five levels of mobility among Brazilian older adults living in the community before and since COVID-19 pandemic (*n* = 1,482) (33).

**LSA levels and life-space**	**Level 1 Home**	**Level 2 Outside home**	**Level 3 Neighborhood**	**Level 4 Town**	**Level 5 Beyond town**	**Total**
**Before pandemic**						
Mean (SD)	7.3 (1.6)	13.9 (4.1)	15.5 (8.5)	18.0 (10.7)	9.1 (10.2)	64.0 (26.0)
Yes (%)	1,459 (98.4)	1,432 (96.6)	1,294 (87.3)	1,320 (89.1)	888 (59.9)	–
No (%)	23 (1.6)	50 (3.4)	188 (12.7)	162 (10.9)	594 (40.1)	–
Frequency (%)						
<1/week	22 (1.5)	42 (2.9)	125 (9.7)	248 (18.8)	559 (63.0)	–
1–3/week	73 (5.0)	123 (8.6)	334 (25.8)	447 (33.8)	218 (24.5)	–
4–6/week	51 (3.5)	92 (6.4)	209 (16.2)	224 (17.0)	49 (5.5)	–
Daily	1,313 (90.0)	1,175 (82.1)	626 (48.4)	402 (30.4)	62 (7.0)	–
Dependency (%)						
Use of assistive devices	52 (3.6)	52 (3.6)	33 (2.6)	31 (2.3)	13 (1.5)	–
Assistance of a person	40 (2.7)	45 (3.2)	54 (4.2)	81 (6.1)	59 (6.6)	–
No use of devices or need of assistance	1,367 (93.7)	1,335 (93.2)	1,207 (93.2)	1,209 (91.6)	816 (91.9)	–
**Since pandemic**						
Mean (SD)	7.3 (1.7)	12.8 (5.1)	7.7 (8.8)	7.9 (9.9)	2.0 (6.3)	37.8 (22.1)
Yes (%)	1,443 (97.4)	1,366 (92.2)	793 (53.5)	728 (49.1)	188 (12.7)	–
No (%)	39 (2.6)	116 (7.8)	689 (46.5)	754 (50.9)	1,294 (87.3)	–
Frequency (%)						
<1/week	15 (1.0)	52 (3.8)	181 (22.8)	244 (33.5)	101 (53.7)	–
1–3/week	66 (4.5)	180 (13.2)	298 (37.6)	316 (43.4)	63 (33.5)	–
4–6/week	62 (4.2)	125 (9.2)	84 (10.6)	46 (6.3)	7 (3.7)	–
Daily	1,300 (87.7)	1,009 (73.9)	230 (29.0)	122 (16.8)	17 (9.0)	–
Dependency (%)						
Use of assistive devices	43 (3.0)	42 (3.1)	12 (1.5)	8 (1.1)	0 (0.0)	–
Assistance of a person	37 (2.6)	32 (2.3)	21 (2.6)	36 (4.9)	14 (7.4)	–
No use of devices or need of assistance	1,363 (94.5)	1,292 (94.6)	760 (95.8)	684 (94.0)	174 (92.6)	–
*p*-value^a^	0.363	<0.001	<0.001	<0.001	<0.001	<0.001

*LSA, Life-Space Assessment; SD, Standard deviation*.

a *Wilcoxon test*.

Table 3 shows mean life-space mobility scores for groups of interest before and since the COVID-19 pandemic, as well as for deltas. *Pardo* (mixed race) individuals had a significantly lower LSA score (*p* < 0.001) before the pandemic compared with white individuals, and this situation persisted since the pandemic (*p* = 0.005). A smaller, but significant reduction in LSA (Δ LSA) (*p* < 0.001) was observed among *pardo* individuals compared with white individuals. A reduction in life-space mobility (Δ LSA) was observed among women compared with men (*p* < 0.008), among older adults aged between 60 and 69 and 70 and 79 years compared with older adults aged 80 years and over (*p* < 0.001), among older people living alone (*p* < 0.001), among individuals with a high educational level (5 or more years of schooling) (*p* < 0.001), and among individuals with a high-income level (four or more minimum wage salaries) (*p* < 0.001).

**Table 3:** Mean life-space mobility scores before and since COVID-19 pandemic and the Δ LSA (before minus since COVID-19 pandemic) according to gender, social determinants, multimorbidity and response to social restriction (*n* = 1,482) (33).

**Characteristic**	**Mean LSA (SD) Before COVID-19 pandemic**	***p*-value^a^**	**Mean LSA (SD) Since COVID-19 pandemic**	***p*-value^a^**	**Mean Δ LSA (SD)**	***p*-value^a^**
Gender		<0.001		<0.001		0.008
Women	62.2 (25.7)		35.0 (20.0)		−27.1 (24.9)	
Men	69.3 (26.3)		46.0 (25.8)		−23.2 (25.2)	
Age group (years)						
60–69	70.1 (24.0)	<0.001	42.8 (22.6)	<0.001	−27.3 (25.8)	<0.001
70–79	63.2 (24.6)	<0.001	36.2 (20.3)	<0.001	−27.0 (24.4)	<0.001
80 and over	43.4 (25.2)	Ref.	23.5 (16.4)	Ref.	−19.9 (22.2)	Ref.
Ethnicity^b^						
White	67.0 (25.1)	Ref.	39.4 (22.9)	Ref.	−27.6 (24.9)	Ref.
Black	64.6 (26.0)	0.389	34.5 (20.5)	0.038	−30.1 (25.2)	0.341
“Pardo”	58.1 (27.0)	<0.001	35.8 (20.7)	0.005	−22.3 (24.7)	<0.001
Marital Status		<0.001		<0.001		0.582
Single/Divorced/Widowed	66.6 (24.5)		40.9 (22.6)		−25.8 (24.9)	
Married	61.0 (27.4)		34.5 (21.1)		−26.5 (25.0)	
Living alone		<0.001		0.476		<0.001
Yes	69.3 (25.9)		39.0 (23.0)		−30.7 (26.5)	
No	63.1 (25.8)		37.9 (22.1)		−25.1 (24.6)	
Educational level		<0.001		<0.001		<0.001
Low (illiterate or 1–4 years of schooling)	50.3 (24.7)		32.5 (20.7)		−17.9 (21.0)	
High (5–8/9 or more)	69.1 (24.7)		39.9 (22.3)		−29.2 (25.7)	
Income (minimum wage salaries) ^c^		<0.001		<0.001		<0.001
<1/2–3	58.6 (25.9)		35.8 (21.1)		−22.9 (23.7)	
4–7/8–10/10 or more	73.0 (23.9)		41.5 (23.4)		−31.5 (26.1)	
Occupation		<0.001		<0.001		0.232
Active	72.6 (24.0)		45.5 (23.3)		−27.1 (27.0)	
Inactive/Unemployed	59.0 (25.9)		33.5 (20.2)		−25.5 (23.7)	
Multimorbidity (two or more) ^d^		<0.001		<0.001		0.656
0–1	69.9 (24.3)		43.5 (23.5)		−26.4 (25.9)	
two or more	59.5 (26.5)		33.7 (20.1)		−25.8 (24.2)	
Social restriction measures		0.822		0.026		0.082
Totally and partially agree and indifferent	64.0 (26.1)		37.7 (22.0)		−26.3 (25.1)	
Totally and partially disagree	64.9 (25.8)		45.0 (25.2)		−19.9 (22.2)	

*LSA, Life-Space Assessment; Δ LSA is the difference in composite scores of LSA before and since pandemic; SD, Standard deviation*;

a *Wilcoxon signed-rank and Kurskal-Wallis test*.

b *“Amarelo” and Indigenous categories were treated as missing due to the low distribution in the sample*.

c *Brazilian minimum wage salary 1,045.00 BRL (corresponding to 189.3 USD; 1st May 2020)*.

d *Multimorbidity included stroke, Parkinson's disease, arthritis, osteoporosis, urinary and fecal incontinence, acute myocardial infarction, intestinal and depressive disease, anxiety, visual and hearing impairment, spine, overweight, hypertension and dizziness*.

Multiple linear regression showed the relationship between Δ LSA and explanatory variables (Table 4). There were significant relationships between Δ LSA and male sex (β = 3.32, 95% CI = 0.33; 6.32), living alone (β = −3.75, 95% CI = −7.09; −0.41), age between 70 and 79 years (β = −4.95, 95% CI = −9.13; −0.78; *ref*. 80 years and over), black race/ethnicity (β= −7.76, 95% CI = −13.14; −2.37; *ref*. pardo), having more than 4 years of schooling (β = 7.94, 95% CI = 4.60; 11.28; *ref*. illiterate or 1–4 years), and having an income of ≥4 minimum wage salaries (β = 4.76, 95% CI = 1.77; 7.75; *ref*. <3 minimum wage salaries). The fit of the regression equation found in the final model was [*F*_(11,1,389)_ = 8.36, *p* < 0.001], *R*^2^ = 0.055.

**Table 4:** Linear regression analyses to identify the association between Δ LSA (Life-space mobility) and gender, social determinants, multimorbidity and response to social restriction measures (*n* = 1,482) (33).

**Characteristic**	**Model crude**		**Model adjusted**	
	**β crude**	**95% IC**	**β adjusted**	**95% IC**
Gender, men (*ref.:* women)	3.92	1.02; 6.81	3.32	0.33; 6.32
Age group (*ref.:* 80 years and over)				
60–69 years	−7.43	−11.07; −3.79	3.11	−7.12; 0.92
70–79 years	−7.14	−11.15; −3.79	–4.95	–9.13; –0. 78
Ethnicity^a^ (*ref:* “*pardo”*)				
White	−5.25	−8.08; −2.41	−1.96	−4.91; 1.00
Black	−7.70	−13.11; −2.29	–7.76	–13.14; –2.37
Living alone	−5.14	−8.46; −1.82	–3.75	–7.09; –0.41
Complete years of schooling > 4 years (ref.: Illiterate or 1–4)	11.30	8.48; 14.11	7.94	4.60; 11.28
Income ≥4 minimum wage salaries (ref.: <3)	8.59	5.99; 11.18	4.76	1.77; 7.75
Occupation active (ref.: inactive/unemployed)	−1.61	−4.25; 1.03	0.57	−2.23; 3.37
Multimorbidity^b^ 0–1 (ref.: two or more)	−0.58	−3.16; −1.99	−1.12	−1.53; 3.78
Social restriction measures (total and partial disagree and indifferent) (ref.: total and partial agree)	6.44	−0.83; 13.70	3.34	−4.00; 10.69

*Adjusted R^2^ = 0.0546; F_11,1389_ = 8.36, df = 11 of 24; p <0.001. β, standardized regression coefficient; CI, confidence interval*.

a *As “Amarelo” and Indigenous categories were treated as missing due to the low distribution in the sample*.

b *Multimorbidity included stroke, Parkinson, arthritis, osteoporosis, urinary and fecal incontinence, acute myocardial infarction, intestinal and depressive disease, anxiety, visual and hearing impairment, spine, overweight, hypertension and dizziness*.

*Model adjusted for sex, age, race, living alone, schooling, income, occupation, multimorbidity, and response to social restriction measures (e.g., lockdown, stay-at-home recommend)*.

## Discussion

Our results showed significant changes in life-space mobility, particularly outside of the home environment (in the neighborhood, in the town, and beyond town). Nearly a third of participants reported that they were completely restricted at home and not receiving visits, and almost half of participants were leaving home only when they needed to get groceries or go to the pharmacy. Regardless of gender and socioeconomic status, participants showed a reduction in their life-space mobility since COVID-19 pandemic. However, reductions in life-space mobility were higher among older people living alone, those aged between 70 and 79 years compared to older people aged 80 years old and over, and black individuals compared to *pardo* individuals, exposing underlying inequalities that might have been aggravated by the pandemic.

We found post-pandemic reductions of around 20 points in LSA scores. A score above 10 points is considered a marker of poor health outcomes (12). Similar ranges of decline in life-space mobility have been associated with future disability in performing activities of daily living (>11.7 points (34), hospital admissions (10.3–22.4 points; (35) and injurious falls (5–24 points; (34, 36). The continuous restriction in life-space mobility due to COVID-19 might increase the risk of developing chronic conditions and functional decline.

We found older adults who were male, who had a moderate to high educational level, and who had a higher income level enjoyed more life-space mobility compared to women, individuals with lower educational and income levels. This can be partly explained by the fact that compared to older women; older men already had greater life-space mobility before the COVID-19 pandemic. Older women are almost twice as likely not to work in comparison to men (37); when working, women are more frequently unpaid, doing activities such as caring for others, home-based work, or domestic chores (38). Previous studies have shown that women's life-space mobility was more frequently restricted (6, 39). Possibly, the life-threatening situation of the COVID-19 pandemic might have not alarmed older men. This is particularly interesting because men were found twice as likely to be at increased risk of severe COVID-19 in all age groups (3). Men were possibly less concerned than women about being contaminated and engaged in more risky activities (40). Societal expectations such as the responsibility of being the family provider, a sense of invulnerability, and misleading messages from the government may have contributed to these behaviors. However, these conjectural explanations may be sample-biased due to the reduced number of men who participated in the current study (26.1%).

Compared with older adults aged 80 and older, participants aged between 70 and 79 years experienced a greater reduction in life-space mobility, but people between 60 and 69 years did not. In our study, among women and men aged 60–69 years old, 42 and 65% reported actively working compared to 24% of women and 40% of men aged between 70 and 79 years (*p* < 0.001). This in part might explain why this age group did not experience substantially reduced life-space mobility. Our data also revealed that multimorbidity was more prevalent among individuals aged 80 and over (92%) and aged between 70 and 79 (86%) compared with individuals aged between 60 and 69 years old (80%; *p* < 0.001). Common reported health conditions that were more prevalent with increasing age were diabetes, hypertension, congestive heart failure, hearing loss, urinary incontinence, and dizziness. Being more vulnerable to severe COVID-19 might have alarmed the oldest individuals and discouraged them from moving around.

Higher reductions in life-space mobility were observed among black individuals compared to older *pardo* individuals. Employment inequalities in this population might in part explain this reduction. Approximately 70% of older adults in Brazil are retired or pensioners, and 15.6% still work to supplement their income (41). Insufficient income for daily expenses is more frequently reported by black (50.3%) and *pardo* (51,1%) older adults in Brazil compared with white older adults (38.6%; (42)), pushing vulnerable populations to seek informal jobs. These jobs were highly restricted during the early months of the COVID-19 pandemic (i.e., informal market, street vending jobs, and domestic jobs; (43) and this situation might have contributed for the reduction of life-space among black individuals.

Systemic disadvantageous conditions, such as high health illiteracy (44), poor health (multiple comorbidities), racism, and poor housing conditions with many people of different generations occupying the same spaces (42) may have influenced how older black people coped with the social and economic restrictions resulting from the pandemic. In our study, black and *pardo* individuals had significantly lower incomes compared to whites (74 vs. 79 vs. 53%; *p* < 0.001, respectively) and were also less educated (illiterate or 1–4 years of schooling: 39 vs. 40.5 vs. 19%; *p* < 0.001, respectively). Low socioeconomic status and physical inactivity during the pandemic combined with underlying health conditions that are common in this population may increase the risk of poor management of non-communicable diseases, disability, and frailty. A population-based study in Brazil showed a worse health pattern for black individuals, with substantially higher prevalence ratios for hypertension, diabetes, stroke, and cognitive decline (42).

Living alone was another social determinant that accounted for a greater restriction in life-space mobility during COVID-19. In Brazil, more than 4.3 million older people were living alone before the pandemic (45), and nearly 60% were women aged between 65 and 74 years (46). In the present study, 85% of the participants who lived alone were women, and nearly 60% were aged between 60 and 69 years. A Brazilian population-based study of 11,967 older adults living alone confirmed a higher prevalence of this household type among women and showed that older people living alone more frequently reported musculoskeletal conditions, hearing loss, falls, and limitations to instrumental activities of daily living (47). Older people living alone are more likely to face emergency department visits and to have general practitioner appointments compared to older adults living with others (48). Unmet basic needs, social isolation and disruption of health services during the COVID-19 pandemic might increase the risk of loneliness, malnutrition, and functional decline (49). Older women in particular are at greater risk of financial abuse and lack of care (50).

Older people with moderate to high educational levels and higher income levels had greater life-space mobility scores before the pandemic compared to the group with lower education and income levels; for them, the impact of the COVID-19 pandemic contributed less to reduction in life-space mobility. These groups were able to appraise health-related information and use resources to adopt shielding strategies. Higher levels of education and social status have been associated with higher health literacy (51). However, it is also argued that higher health literacy scores are associated with lower fear of COVID-19 and lower likelihood of depression (51), which may have influenced better educated older people to take risks. Having a private vehicle for transportation and access to locations outside urban centers might have increased the areas wealthy older people moved around. Socioeconomic inequalities from birth onward favor better health trajectories for individuals with higher educational and income levels, and these gaps commonly increase with age (52).

The implications of our findings are 2-fold. First, our results underline the need to structure urgent comprehensive responses to mitigate pandemic consequences among older adults living alone, among black individuals, and for people with lower income and education levels. Prioritized actions should be set up urgently to assist these vulnerable groups in the community, strengthening existing policies in the public sector, particularly the Family Strategy Program in the National Health Service (or SUS). In Brazil, the older population (more than 80%) largely relies on public health care, and this percentage is even higher among Afro-Brazilians and the poor (53). Integrated person-centered care can include life-space assessment and monitoring over time, helping service providers and health care teams capture short- and long-term functional consequences of the pandemic. The provision of long-term care services at the national and subnational levels should also be envisioned.

Second, innovative digital technologies should be envisioned to scale up best-buy interventions, such as digital platforms to deliver physical activity and rehabilitation programs (54). Mobile apps that can track life-space mobility over time, creating alerts for unusual reductions, are promising resources (55, 56). Digital technologies are increasingly important strategies for engaging older people and for providing access to a wide range of services. The use of digital technology has been increasing annually among older people. In Brazil, the proportion of older adults who access the Internet has increased from 24.7% in 2016 to 31.1% in 2017 (57), and about 80% of households in the Southeast region have Internet access (58). However, digital illiteracy and high costs to purchase mobile phones with internet connection packages are still a greater barrier, affecting the ability of low-income older people to use services that are being required during the pandemic (59). Public-private partnerships can ensure that services are available to these groups to prevent further aggravation of health inequities during COVID-19 pandemic.

The results of the present analysis have some limitations. Some geographical regions of Brazil were less represented, such as the south and central regions. However, the southeast region, which is the most populated and contains a higher proportion of older people, is well-represented in our sample. Although we made efforts to reach vulnerable older adults in some urban communities (i.e., slums), these areas may be underrepresented, but face-to-face interviews were unsafe for both participants and researchers during the COVID-19 pandemic. Recall bias is also possible, since the participants self-reported their life-space mobility conditions before COVID-19. We also cannot assume that the restriction in life-space mobility that we observed is solely related to the pandemic. Timing and intensity of the pandemic might have influenced the reductions in life-space mobility. Furthermore, we used a broad and general question to capture adherence behavior to stay-at-home and social distancing recommendations. That question alone might not be able to capture all older adults' views and experiences during the pandemic.

Mobility concerning life spaces includes not only walking, but also other modes of transport (e.g., subway, train, private vehicle, or bus), particularly for moving beyond one's neighborhood (town and beyond town zones). Future studies should specifically address restrictions on transportation during the pandemic, which may have varied according to the sizes of cities, population density, and regulatory policies determined by local governments to deal with coronavirus transmission. Environmental barriers and enablers inside the house and in the community also require further studies. This study focuses on the first wave of a cohort study and is cross-sectional, which limited causal relations and the determinations of trajectories of life-space mobility for different groups. We believe that the results of a 12-month follow-up will help to better understand the short- and long-term impacts of the COVID-19 pandemic on life-space mobility.

Social restriction measures due to a pandemic caused substantial limitations in older adults' life-space mobility in Brazil. Social inequalities should be recognized, and concerted action should be taken to overcome the deterioration in life-space mobility among the most vulnerable groups of older people. Worldwide, failure to minimize health inequalities—amplified by the pandemic—may jeopardize the desired achievements of the Decade of Healthy Aging (60).

## Data Availability Statement

The datasets presented in this study can be found in online repositories. The names of the repository/repositories and accession number(s) can be found in the article/supplementary material.

## Ethics Statement

The studies involving human participants were reviewed and approved by Ethics Committee of Universidade Cidade de São Paulo (protocol number 4.032.523). The ethics committee waived the requirement of written informed consent for participation.

## Author Contributions

MP participated in concept design, data collection, data analysis, interpretation, drafting, critical revision, and approval of the article. CL, ML, and RS participated in concept design, data collection, data analysis, interpretation, critical revision, and approval of the article. JA participated in data collection, data analysis, interpretation, critical revision, and approval of the article. AS, PP, FT-S, DP, and PB participated data collection, interpretation, critical revision, and approval of the article. ED participated interpretation, critical revision, and approval of the article. All authors contributed to the article and approved the submitted version.

## Cansort SCI Affiliations

Adriana Guedes Carlos, Professor Aurelio Dias Santos, Professor Etiene Oliveira da Silva Fittipaldi, Professor Hércules Campos, Professor Juliana Maria Gazzola, Dr. Mirian Moreira, Professor Mônica Beatriz Ferreira, Nayara Tasse de Oliveira Cirino, Professor Renata Oliveira Dantas, Renata dos Ramos Varanda, Professor Suzana Albuquerque de Moraes, Professor Guilherme Medeiros de Alvarenga, Professor Cristina Cristovão Ribeiro da Silva, Sarah Giulia Bandeira Felipe and Professor Lygia Paccini Lustosa (*in memoriam*).

## Conflict of Interest

The authors declare that the research was conducted in the absence of any commercial or financial relationships that could be construed as a potential conflict of interest.
